# Heavy metals in fish and its association with autoimmunity in the development of juvenile idiopathic arthritis: a prospective birth cohort study

**DOI:** 10.1186/s12969-019-0344-3

**Published:** 2019-07-02

**Authors:** Erik Kindgren, Carlos Guerrero-Bosagna, Johnny Ludvigsson

**Affiliations:** 1 Department of Pediatrics, Västervik Hospital, Västervik, Sweden; 2 Division of Pediatrics, Department of Clinical and Experimental Medicine, Linköping University, Linköping, Sweden; 3 Department of Pediatrics, Skaraborg Hospital, SE-541 85 Skövde, Skövde, Sweden; 4 Avian Behavioural Genomics and Physiology Group, IFM Biology, Linköping University, Linköping, Sweden; 5 Crown Princess Victoria Children’s Hospital, Region Östergötland, Linköping, Sweden

**Keywords:** Juvenile idiopathic arthritis (JIA), Arthritis, Epidemiology, Autoimmunity, Nutrition, Fish, Aluminium, Cadmium, Lithium, Heavy metals, Rheumatic disease

## Abstract

**Background:**

The etiology of Juvenile Idiopathic Arthritis (JIA) is poorly understood. The purpose of this study was to examine the possible influence of early nutrition on later development of JIA.

**Methods:**

In a population-based prospective birth cohort of 15,740 children we collected nutritional data, including fish consumption, and biological samples during pregnancy, at birth and at different ages. 16 years after study inclusion we identified 42 children with JIA, of whom 11 were positive for Antinuclear Antibodies (ANA). Heavy metals were analysed in cord blood of all 42 JIA patients and 40 age and sex-matched controls. A multivariable logistic regression model, adjusted for relevant factors, was used as well as Mann-Whitney U-test.

**Results:**

Fish consumption more than once a week during pregnancy as well as during the child’s first year of life was associated with an increased risk of JIA (aOR 4.5 (1.95–10.4); *p* < 0.001 and aOR 5.1 (2.1–12.4) *p* < 0.001) and of ANA-positivity (aOR 2.2 (1.4–3.6); *p* = 0.002 and *p* < 0.001). Concentrations of Al, Cd, Hg and Li in cord blood were significantly higher in the JIA-group than in controls. The ANA-positive, all of whom had consumed fish >once/week their first year, had significantly higher concentrations of Al (*p* < 0.001), Cd (*p* = 0.003), and Li (*p* < 0.001) in cord blood than controls. Frequency of fish consumption correlated with concentrations of Cd (*p* = 0.003), Li (*p* = 0.015) and Hg (*p* = 0.011).

**Conclusions:**

Moderate exposure to heavy metals, associated with fish consumption, during pregnancy and early childhood may cause effects on the immune system of the offspring, resulting in ANA positivity and JIA.

## Introduction

Juvenile idiopathic arthritis (JIA) is the most common chronic childhood rheumatic disease. It is a heterogeneous autoimmune disease comprising seven categories with onset before 16 years of age [1]. Antinuclear antibody (ANA) positivity can be found in all subtypes of JIA but is more frequent in patients with oligoarthritis and rheumatoid factor (RF) negative polyarthritis [2, 3]. The starting point of autoimmunity which leads to JIA and Rheumatoid Arthritis (RA) is still unknown. These autoimmune diseases are severe, even disabling, and incurable. Therefore, it is important to try to prevent their development, both for the patients themselves and for society.

We have performed a prospective study focusing on environmental factors during pregnancy and childhood in a general population. The aim is to explore environmental factors, such as early feeding, in relation to risk of later development of JIA. Breastfeeding seems to be associated with JIA, as described in a previous paper [4]. Increased knowledge of the role of early nutrition and its association with autoimmunity is of crucial importance, as dietary recommendations may help to prevent these chronic diseases.

## Material and methods

The Swedish National Patient Register, launched in 1964, is maintained by the Swedish National Board of Health and Welfare (http://www.socialstyrelsen.se/english). More than 99% of all somatic and psychiatric hospital discharges, as well as outpatient visits from both private and public caregivers, are recorded in this population-based register. Several items are recorded, including International Classification of Diseases (ICD) codes and the personal identity number (PIN - a unique 10-digit number assigned to all Swedish residents) [5].

The Swedish JIA-registry started in 2009. In 2014, there were 1700 patients included and coverage was almost complete. Through the unique PIN, information on each individual patient can be linked to other registers.

In Sweden, all children aged 0–18 years diagnosed with JIA or RA are treated at paediatric clinics in hospitals or at paediatric rheumatology clinics.

### Participants and design

The current study was part of the All Babies in Southeast Sweden-project (ABIS), which aims to study causes of immune-mediated diseases by following a general population birth cohort though childhood and adolescence. All parents with children born between Oct 1st 1997 and Oct 1st 1999 in Southeast Sweden (*n* = 21,700) were asked to participate in the study. 17,055 (78.6%) of the families gave their informed consent to participate. According to the ABIS-protocol the children were monitored by biological samples taken at birth and then at the ages 1, 2.5, 5, 8, 10–12 and 13–16 years as well as questionnaires about life-style and environmental factors. A detailed diary was used for daily registration of certain facts related to nutrition and infections including the exact time (date) of introduction of different food items during the first year of life. The 1-year questionnaires were completed by 10,883 families, and in addition, diaries were collected for 9849 children. All subjects with missing values were excluded from the statistical model.

**Fig. 1:**
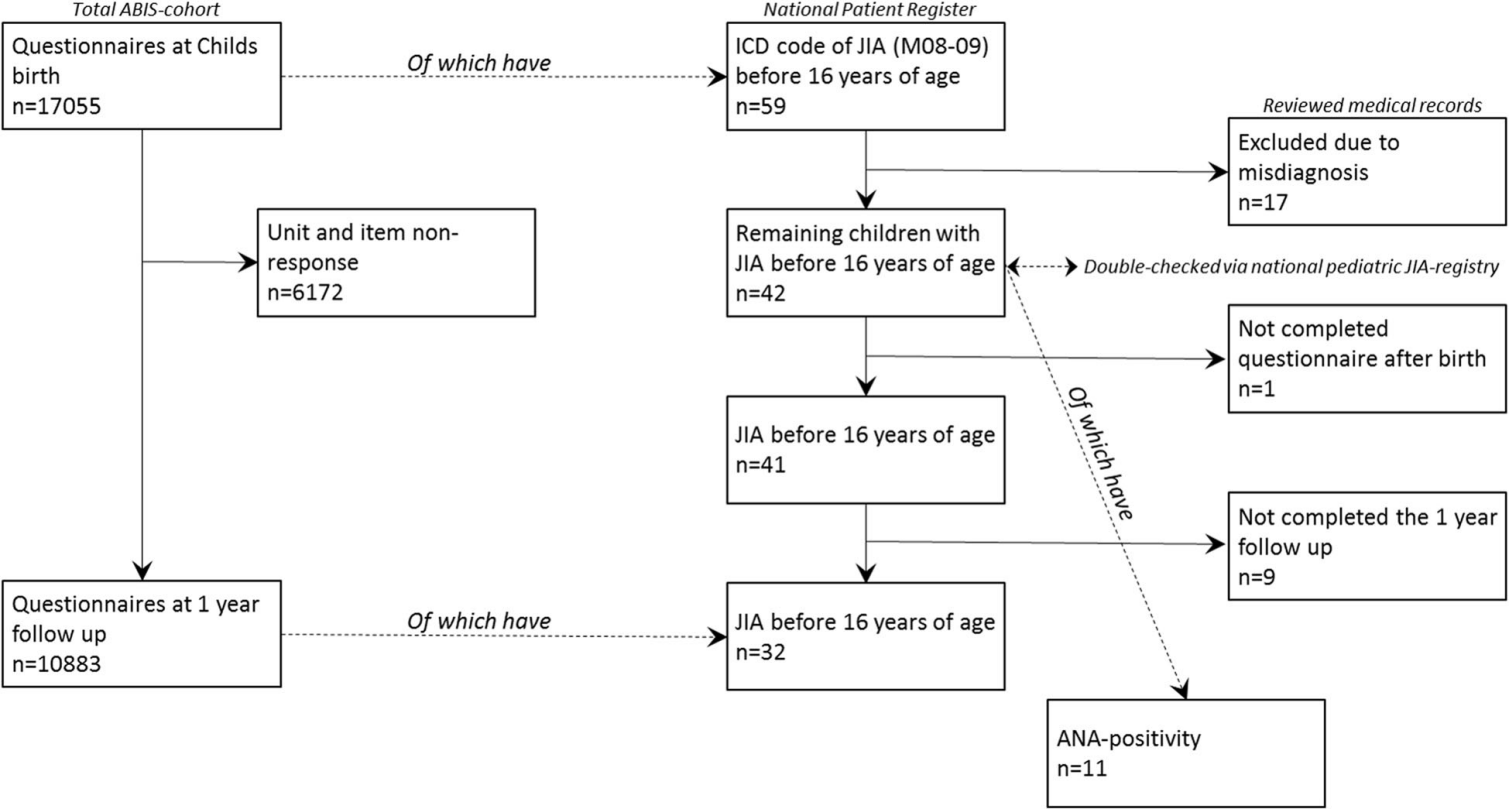
Flow-chart of the study sample, showing the number of total participants, patients with Juvenile Idiopathic Arthritis (JIA) and Antinuclear antibodies (ANA). ICD, International Classification of Diseases

Every member of the ABIS-cohort was followed to see if they developed JIA by linking data from the ABIS-cohort and The Swedish National Patient Register [6] by using a unique 10-digit PIN [5]. We identified 59 children with an ICD code of JIA (ICD 9–10 code M08–09) and had accepted to participate in ABIS (see Fig. 1). Three of children had moved out of the ABIS region, but could be found and included with the PIN. 17/59 patients were excluded due to misdiagnosis (mostly monoarthritis that later proved to be reactive arthritis), after communication with the paediatric rheumatologists at local hospitals who reviewed the medical records of all 59 patients. Of the remaining 42, had 41 completed the questionnaire after delivery, and 32 of them had completed both the questionnaire after delivery and the 1 year follow up questionnaire. 29 of 32 had also completed the detailed nutrition diary during the child’s first year. All cases of JIA and their categories were double-checked via The Swedish paediatric JIA-registry. ANA positivity was detected in 11 patients with JIA. Data on fish consumption were collected from both the detailed diary and the questionnaires. Heavy metals were analysed in all 42 JIA patients and in an additional 40 randomly selected age and sex-matched controls.

### Serology

ANA positivity was detected using indirect immunofluorescence with human epithelioid cells (Hep-2 cells) as substrate [7]. ANA positivity was defined as at least two positive results (titer of ≥1:160) analysed at least 3 months apart. RF was determined by latex agglutination test.

### Cord blood analyses

The analysis of heavy metals was performed by ALS Scandinavia AB (Luleå, Sweden) using the ‘ultrasensitive inductively coupled plasma sector field mass spectrometry method’ (ICP-SFMS) [8] after acid digestion with HNO3, according to the standards ISO 17294-1 (https://www.iso.org/standard/32957.html) and 2 (https://www.iso.org/standard/62962.html), and the EPA Method 200.8 (https://www.epa.gov/homeland-security-research/epa-method-2008-determination-trace-elements-waters-and-wastes). Heavy metals analysed were: Aluminium (Al), Cadmium (Cd), Mercury (Hg), Lithium (Li), Lead (Pb), Zinc (Zn), Copper (Cu), Arsenic (As), Magnesium (Mg) and Iron (Fe).

### Statistics

Homogeneity of variance was tested using Levene’s test and the normality for independent variables was revised both graphically and by the Shapiro–Wilk test.

Odds ratio (OR) with 95% confidence intervals (95% CIs) were estimated using (univariate) logistic regression. JIA and ANA positivity were used as the dependent variables. Amount of dietary fish intake and possible confounding factors (listed below) associated with incidence of JIA were analysed. Dietary fish intake was also dichotomized to either low intake (< 1 per week) or moderate-high intake (≥1 per week). At the time when ABIS was initiated, the Swedish National Institute of Public Health recommended dietary intake of fish 2–3 times a week during pregnancy and 2–3 times a week during the first year of life. Variables with *p* < 0.1 in the univariate analyses were included in the final multivariate logistic regression model as possible confounding factors associated with fish consumption.

Independent samples t-test and Mann-Whitney U-test, adjusted for confounding factors (listed below), was used to calculate differences in levels of heavy metals and fish consumption between the groups. All *P*-values are two-tailed and a value below 0.05 and a 95% CI not overlapping the null value 1.00 for the OR were considered as statistically significant.

Possible confounding factors included heredity for rheumatism (JIA or RA in first- and second-degree relatives), parity, mode of delivery, preterm birth, duration of (exclusive and total) breastfeeding, introduction of formula, parental age, education, smoking habits, and whether or not the parents were born outside Sweden.

Statistics were calculated using the Statistical Package for Social Science (SPSS 25.0 software; SPSS Inc., Chicago, IL, USA).

## Results

### Cases

Forty-one cases of JIA with complete questionnaires after delivery were identified. The number and proportion of patients in each JIA category are shown in Table 1. The unclassified category based on the ILAR-criteria [1], either did not fulfil criteria for any category or they met categorization criteria for 2 subtypes. Family history of RA/JIA in first- and second-degree family member are presented in Table 1.

**Table 1:** Risk of Juvenile Idiopathic Arthritis (JIA) and Antinuclear antibodies (ANA) according to dietary fish intake and family history. OR and *p*-values from logistic regression. Classification according to ILAR criteria. The number and proportion of patients in each category

	Controls	JIA			ANA+		
		Multivariable logistic regression model analysis vs controls §					
			aOR (95% CI)	*p*		aOR (95% CI)	*p*
Fish more than once a week during pregnancy	(*n* = 15,730)	(*n* = 41)			(*n* = 10)		
All fish	4764 (31%)	19 (46%)	**4.5 (1.9–10.4)**	**< 0.001**	6 (60%)	**2.2 (1.4–3.6)**	**0.002**
Fish from Baltic Sea	791 (6%)	4 (10%)	**2.3 (1.1–5.0)**	**0.034**	3 (30%)	**7.6 (1,9–30.2)**	**0.001**
Fish from lake	255 (2%)	0 (0%)	0	0.74	0 (0%)	0	1.000
Other fish	4042 (26%)	17 (42%)	**2.6 (1.3–5.1)**	**0.009**	5 (50%)	5.5 (0.8–14,7)	0.085
Fish more than once a week during the baby’s first year	(n = 10,473)	(*n* = 31)			(*n* = 8)		
All fish	4547 (43%)	24 (77%)	**5.1 (2.1–12.4)**	**< 0.001**	**8 (100%)**	*	**0.001**
Fish from Baltic Sea	1582 (15%)	9 (29%)	**2.3 (1.1–5.1)**	**0.035**	4 (50%)	**5.6 (1.4–22.6)**	**0.014**
Fish from lake	458 (4%)	1 (3%)	0.7 (0.097–5.2)	0.74	0 (0%)	0	1.000
Other fish	3387 (32%)	17 (55%)	**2.9 (1.4–5.9)**	**0.005**	5 (63%)	**1.7 (1.1–2.7)**	**0.016**
Family history / Heredity for JIA/RA							
Mother with RA (n)	157 (1%)	1 (2%)	2.5 (0.3–18.5)	0.362			
Father with RA (n)	64 (0%)	0 (0%)	0	1.000			
Siblings with JIA/RA (n)	48 (0%)	0 (0%)	0	1.000			
2nd degree family member with RA (n)	1431 (9%)	8 (20%)	**1.3 (1.0–1.5)**	**0.022**			
Category at Onset (ILAR) (n)							
Oligoarticular†		19 (46%)					
Polyarticular‡		5 (12%)					
Enthesitis-related arthritis		4 (10%)					
Psoriatic arthritis		4 (10%)					
Systemic		5 (12%)					
Unclassifiable		4 (10%)					

§heredity for rheumatism (JIA or RA in first- and second-degree relatives), parity, mode of delivery, preterm birth, duration of (exclusive and total) breastfeeding introduction of formula, mother’s civil status, parental age, education, smoking habits, and whether or not the parents were born outside Sweden

*Logistic regression not possible. *P*-value from Chi-square, statistically significant *p*-values and OR in bold

†Patients with oligoarticular disease, not possible to classify as extended or persistent because of missing data

‡Patients with polyarticular disease, not possible to classify as RF positive or negative because of missing data

### Fish and JIA

**Fig. 2:**
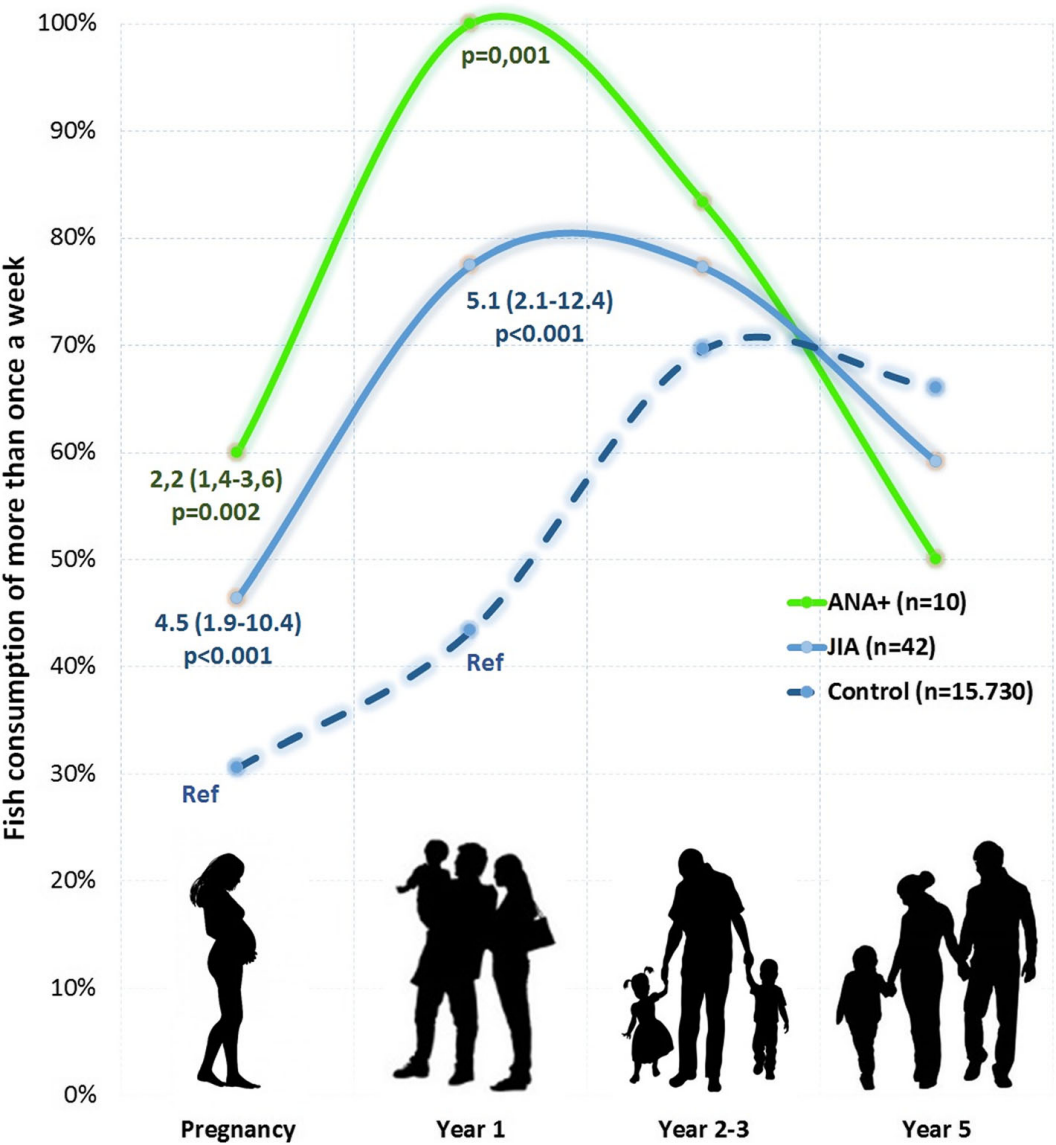
Fish consumption of more than once a week during pregnancy as well as during the child’s first years of life. Odds Ratio and p-value from Logistic regression

Fish consumption more than once a week during pregnancy was associated with an increased risk of JIA (aOR 4.5 (1.95–10.4); *p* < 0.001). The child’s consumption of fish more than once a week during the first year of life was also associated with an increased risk of JIA (aOR 5.1 (2.1–12.4) p < 0.001), as illustrated in Table 1 and Fig. 2. 87% (27/31) of children with JIA was exposed to fish more than once a week during pregnancy or during their first year, compared to 56% in the general population (aOR 5.4 (1.9–15.4) *p* = 0.002).

### Fish and ANA

Fish consumption more than once a week during pregnancy as well as during the child’s first year of life was associated with an increased risk of ANA-positivity (aOR 2.2 (1.4–3.6); p = 0.002 and p < 0.001, respectively). 100% of children with ANA-positivity had consumed fish more than once a week during their first year. Fish consumption at the age of 2.5 and 5 years showed no association with ANA-positivity (Table 1 and Fig. 2).

### Heavy metals and ANA

Concentrations of Al, Cd and Li in cord blood were significantly higher in the ANA-group than in controls (Table 2). Chi^2^-test with concentrations of heavy metals dichotomized below/above the detection level (Al 5.00 μg/l; Cd 0.05 μg/l; Li 1.00 μg/l) showed significant results for Al (*p* = 0.040), Cd (*p* = 0.008) and Li (*p* = 0.006). 82% of the children with ANA antibodies had concentrations of Al, Cd, or Li higher than the mean, and 64% had concentrations two standard deviations above the mean. 91% (10/11) of the ANA-positive had elevated concentrations higher than the mean of one or more of all the analysed heavy metals.

**Table 2:** Concentrations of heavy metals in cord blood. *P*-values, versus controls, from independent samples t-test and Mann-Whitney U-test. Dietary fish intake during pregnancy, dichotomized to either low intake (< 1 per week) or moderate-high intake (≥1 per week). *P*-values from independent samples t-test

						Fish consumption		
	Controls	JIA	*p*	ANA+	*p*	< 1 per week	≥ 1 per week	*p*
	(*n* = 40)	(*n* = 42)		(*n* = 11)		(*n* = 49)	(*n* = 30)	
Aluminium, Al (μg/L)								
Mean (SD)	6.1 (4.8)	11.2 (9.7)	**< 0.001**	15.4 (12.9)	**< 0.001**	8.1 (7.3)	10.1 (9.6)	0.261
Median (Range)	5.0 (5.0–34.0)	7.0 (5.0–47.2)		12.1 (5.0–47.2)		6.0 (5.0–41.4)	7.0 (5.0–47.2)	
Cadmium, Cd (μg/L)								
Mean (SD)	0.07 (0.08)	0.19 (0.50)	**< 0.001**	0.18 (0.30)	**< 0.001**	0.11 (0.19)	0.16 (0.45)	**0.003**
Median (Range)	0.05 (0.05–0.47)	0.07 (0.05–3.12)		0.08 (0.05–1.08)		0.07 (0.05–1.08)	0.07 (0.05–3.12)	
Mercury, Hg (μg/L)								
Mean (SD)	0.24 (0.08)	0.33 (0.14)	**< 0.001**	0.33 (0.17)	0.067	0.28 (0.13)	0.31 (0.11)	**0.011**
Median (Range)	0.20 (0.20–0.53)	0.30 (0.20–1.01)		0.29 (0.20–0.65)		0.21 (0.20–1.01)	0.30 (0.20–0.65)	
Lithium, Li (μg/L)								
Mean (SD)	1.01 (0.04)	2.79 (6.03)	**< 0.001**	6.21 (11.41)	**< 0.001**	1.31 (0.70)	3.01 (7.14)	**0.015**
Median (Range)	1.00 (1.00–1.22)	1.00 (1.00–39.40)		2.08 (1.00–39.40)		1.00 (1.00–4.14)	1.00 (1.00–39.40)	
Lead, Pb (μg/L)								
Mean (SD)	0.64 (0.50)	0.52 (0.06)	0.902	0.53 (0.08)	0.952	0.58 (0.44)	0.56 (0.18)	0.790
Median (Range)	0.5 (0.5–3.5)	0.5 (0.5–0.8)		0.5 (0.5–0.8)		0.5 (0.5–3.5)	0.5 (0.5–1.3)	
Zinc, Zn (μg/L)								
Mean (SD)	1070 (521)	1039 (256)	0.412	1184 (375)	0.216	1061 (418)	1062 (404)	0.998
Median (Range)	966 (683–3600)	984 (665–1780)		1030 (707–1780)		996 (683–3600)	960 (665–1062)	
Copper, Cu (μg/L)								
Mean (SD)	417 (106)	509 (281)	0.516	490 (171)	0.291	473 (239)	512 (286)	0.997
Median (Range)	408 (257–579)	432 (233–1540)		425 (291–866)		424 (233–1460)	489 (259–1540)	
Arsenic, As (μg/L)								
Mean (SD)	1.09 (0.27)	2.04 (1.96)	0.053	1.45 (0.95)	0.112	1.88 (1.77)	1.86 (1.90)	0.957
Median (Range)	1.0 (1.0–1.9)	1.0 (1.0–9.3)		1.0 (1.0–4.2)		1.0 (1.0–9.3)	1.0 (1.0–7.9)	
Magnesium, Mg (mg/L)								
Mean (SD)	16.6 (1.9)	16.9 (2.6)	0.780	17.0 (4.0)	0.944	16.6 (1.9)	17.4 (2.7)	0.223
Median (Range)	16.7 (12.7–19.4)	16.8 (10.5–26.4)		16.7 (10.5–26.4)		16.7 (12.3–19.8)	17.3 (12.6–26.4)	
Iron, Fe (mg/L)								
Mean (SD)	2.97 (1.70)	2.44 (1.58)	0.450	2.65 (2.76)	0.275	2.67 (1.30)	2.43 (1.94)	0.598
Median (Range)	2.2 (1.6–6.7)	2.1 (0.0–10.4)		1.8 (0.0–10.4)		2.2 (1.2–6.7)	2.0 (0–10.4)	

Statistically significant *p*-values in bold

### Heavy metals and JIA

Concentrations of Al, Cd, Hg and Li in cord blood were significantly higher in the JIA-group than in controls (Table 2). Chi^2^-test of heavy metals dichotomized below/above the detection level (Al 5.00 μg/l; Cd 0.05 μg/l; Hg 0.200 μg/l; Li 1.00 μg/l) showed significant results for Al (*p* < 0.001), Cd (*p* < 0.001), Hg (*p* < 0.001) and Li (*p* < 0.001). 71% of the children with JIA had concentrations of Al, Cd, Hg or Li higher than the mean, and 52% had concentrations two standard deviations above the mean. 95% (40/42) in the JIA-group had elevated concentrations higher than the mean of one or more of all the analysed heavy metals.

### Heavy metals and fish

Frequency of (total) fish consumption during pregnancy correlated with concentrations of Cd (*p* = 0.003), Li (*p* = 0.015) and Hg (*p* = 0.011) in cord blood, but not with concentrations of Al (*p* = 0.261) (Table 2). Consumption of fish from the Baltic Sea correlated with concentrations of Cd (p = 0.011), and consumption of “other fish” (defined as all sorts of fish except fish from the Baltic Sea and fresh water) correlated with concentrations of Cd (*p* = 0.028), Li (*p* = 0.006) and Hg (*p* = 0.012). Frequency of consumption of fish from fresh water was extremely low during pregnancy, as shown in Table 1. All statistical associations remained statistically significant when potential confounders (described in Material and methods section) were included in the model.

## Discussion

In a population-based prospective birth cohort survey, we found that consumption of fish during pregnancy and the child’s first year of life was associated with an increased risk of ANA-positivity and JIA. Concentrations of Cd, Hg and Li in cord blood correlated with the amount of fish the pregnant woman had eaten. Concentrations of Al, Cd, Hg and Li in cord blood were also significantly higher in the JIA-group than in controls. The ANA-positive, all of whom had consumed fish >once/week their first year, had significantly higher concentrations of Al, Cd and Li in cord blood than controls.

It could be hypothesized that eating fish may reduce the risk of inflammatory diseases because it is a major source of long-chain polyunsaturated fatty acids (PUFAs), especially n-3 PUFAs and micronutrients that may have anti-inflammatory properties and may modulate immune responses [9–11]. Gestational exposure to n-3 PUFAs is well known to have beneficial effects on children’s subsequent health [12] On the other hand, fish is contaminated by marine pollutants, mainly PCBs, dioxins and other polychlorinated compounds, and heavy metals [13, 14]. The positive effect of these PUFAs may therefore in part counterbalance the toxic impact of contaminants.

Murphy et al. have suggested that cadmium inhalation could be a plausible trigger for RA [13]. Smoking is highly associated with RA [12, 14]. Cigarette smoke is an important environmental cause of elevated cadmium levels in humans [14]. Smokers have been observed to have blood cadmium concentrations twice as high as non-smokers [9]. Moreover, increased cadmium exposure has been reported in non-smoking men with RA [10]. Hair cadmium levels are 3-fold higher in RA patients with occupational exposure to cadmium, irrespective of smoking history [15]. Patients with RA are more likely to have a profession associated with exposure to cadmium [14]. In addition, cadmium has been detected in the synovial fluid of patients with RA [16].

The main source of cadmium exposure in non-smokers is the diet [17]. Intestinal absorption of dietary cadmium is increased in individuals with low iron stores, and significantly higher levels of cadmium could be found in women of reproductive age than in age-matched men [18]. Iron and cadmium compete with one another for transport into the intestinal mucosal cells. Since in pregnancy, the absorption of micronutrients increases, cadmium absorption is also increased [19, 20]. Generally, cadmium concentration in fish was higher in samples taken on the Baltic coast (Baltic Sea) compared to samples from the Swedish west coast [21]. Cadmium in low concentrations has been associated with a pro-inflammatory state [22], and high-dose cadmium given to rats has been shown to intensify disease activity of collagen-induced arthritis and expression of cytokines [23]. Several other studies have showed that cadmium is not only toxic to living organisms, but may also acts as an immunomodulator [24–26].

Humans do not have an effective elimination pathway for cadmium, and as a consequence the half-life of cadmium in the body is estimated to be 15–20 years [27]. Massive cadmium accumulation in the body often leads to conditions such as respiratory disease, kidney failure, neurological disorders, and occasionally death [28]. Pharmacokinetic studies have shown that cadmium does not easily reach the foetus, but it accumulates in the placenta in high concentrations [29, 30]. Although there is evidence that adult exposure to cadmium causes changes in the immune system, there are few reports of immunomodulatory effects of prenatal exposure to cadmium. Hanson et al. suggest that even very low levels of cadmium exposure during pregnancy can result in long term detrimental immunomodulatory effects in mouse offspring [30].

Aluminium is a potent stimulator of the immune system, which is the very reason it is used as an adjuvant [31]. In the absence of Al most vaccinations fail to launch an adequate immune response [31, 32]. Pro-inflammatory cytokines, such as interleukin-1 and interleukin-6, are needed for adequate stimulation of antibody-producing B-cells. These cytokines are induced by Al adjuvants in vaccines [33]. Or results showed that cord blood aluminium does not correlate with fish consumption, but correlate with development of JIA. We have no explanation for this results. One can speculate about the source of the aluminium that leads to higher cord blood levels of Aluminium in JIA patients. Certain families may have been cooking in aluminium containers more often than other families. Another source could be intake of aluminium from foods (other than fish) or consumed by using aluminium-containing pharmaceuticals.

Mercury-induced autoimmunity in mice is an established model to study the mechanisms of the development of ANA. Immunotoxic effects, including ANA-positivity, have been clearly demonstrated in murine models in response to both organic and inorganic mercury [34]. In humans, occupational mercury exposure (as a result of small-scale gold mining) has been associated with elevated serum titers of ANA [11]. Methylmercury, at low levels generally considered safe, was associated with increased risk of high-titer ANA positivity among reproductive-age females in the general U.S. population [35].

Inorganic mercury contaminates waterways, can be biotransformed to methylmercury, and bioaccumulate in fish. Consumption of methylmercury-laden fish represents the most common route of exposure for humans [36]. Generally, mercury concentrations in fish living in the Baltic Sea are above the suggested target level for concentrations for the protection of predators against secondary poisoning of 20 ng/g wet weight [21].

Lithium therapy has been acknowledged to induce thyroid autoimmunity. Studies have reported a high prevalence of antithyroid antibodies in patients with affective disorders receiving lithium [37, 38]. Moreover, older studies have shown an increase in ANA-positivity amongst patients with affective disorders on lithium therapy [39–41].

Thus, it is reasonable to believe that the increased concentrations of both cadmium, aluminium, mercury and lithium may play a role in changes in the immune balance, which could contribute to the development of autoimmune rheumatic diseases.

### Strengths and limitations of the study

The small patient sample is a weakness due to the nature of the study, i.e. a birth cohort of a relatively rare disease. Different categories of JIA should be studied as separate groups [42, 43], but our study sample was too small to allow subgroup analysis. Although there were dropouts, these were not found to associate with heredity or subsequent diagnosis; therefore, it seems unlikely that our findings are a result of skewed attrition due to lost to follow-up participants. Other possible limitations are unmeasured contaminants in the fish, which may be associated with heavy metals, and leading to these observations, as well as unmeasured confounders, misclassification of diagnosis, selection bias and generalizability of the data.

Our study has some notable strengths. First, the prospective study design avoids the potential recall and selection biases of retrospective studies that collect data on nutrition and lifestyle after the diagnosis. The prospective design distributes any possible measurement biases equally between the JIA cases and non-cases, and recall bias is not likely to explain our findings. The ABIS cohort has been found to be representative for the Swedish general population in aspects such as education level [44]. Second, all cases of JIA were collected through the unique 10-digit PIN with codes from the Swedish National Patient Register with more than 99% coverage of all visits from both private and public caregivers. Subsequently, all cases were confirmed. This is a significant advantage over studies that rely only on self-report. Third, we have validated all diagnoses via The Swedish paediatric JIA-register and with the local physicians/hospitals. Fourth, the access to detailed information on important early life factors allowed us to control for several potential confounding factors that may have influenced our observed associations.

## Conclusions

In conclusion, our results indicate that consumption of fish during pregnancy and first year of life may be a risk factor for development of JIA. Heavy metals could trigger autoimmunity leading to JIA, but further studies are needed to explore the possible causal link between heavy metal diet in early life (included pregnancy) and development of JIA. Our findings suggest that even moderate exposure to aluminium, cadmium, mercury and lithium during pregnancy and early childhood may cause effects on the immune system of the offspring, resulting in ANA positivity and JIA. Exposure to heavy metals during pregnancy and early childhood should be limited, and heavy metal content should be considered for inclusion in future guidelines for food and nutrition during pregnancy and infancy. More knowledge of the role of the child’s early nutrition and its association to autoimmunity is of importance, as future guidelines for food and nutrition during pregnancy and infancy may help to prevent this chronic disease.

## Data Availability

The datasets used and analysed during the current study are available from the corresponding author on request.

## Abbreviations

ABIS: All Babies in Southeast Sweden
Al: Aluminium
aOR: Adjusted odds ratio
As: Arsenic
Cd: Cadmium
CI: Confidence Interval
Cu: Copper
Fe: Iron
Hg: Mercury
ICD: International Classification of Diseases
ILAR: International League of Associations for Rheumatology
JCA: Juvenile Chronic Arthritis
JIA: Juvenile Idiopathic Arthritis
JRA: Juvenile Rheumatoid Arthritis
Li: Lithium
Mg: Magnesium
OR: Odds Ratio
Pb: Lead
PIN: Personal Identification Number
RA: Rheumatoid Arthritis
RF: Rheumatoid Factor
RR: Relative risk
Zn: Zinc
